# The functional relevance of visuospatial processing speed across the lifespan

**DOI:** 10.1186/s41235-023-00504-y

**Published:** 2023-08-04

**Authors:** Courtney Aul, Julia M. Brau, Alexander Sugarman, Joseph M. DeGutis, Laura T. Germine, Michael Esterman, Regina E. McGlinchey, Francesca C. Fortenbaugh

**Affiliations:** 1 Boston Attention and Learning Laboratory (BALLAB), VA Boston Healthcare System, Boston, MA USA; 2 National Center for PTSD, VA Boston Healthcare System, Boston, MA USA; 3 Translational Research Center for TBI and Stress Disorders (TRACTS), VA Boston Healthcare System, Boston, MA USA; 4 Department of Psychiatry, Harvard Medical School, Boston, MA USA; 5 Institute for Technology in Psychiatry, McLean Hospital, Belmont, MA USA; 6 Department of Psychiatry, Boston University Chobanian and Avedisian School of Medicine, Boston, MA USA

**Keywords:** Visual speed of processing, Visual attention, Lifespan trajectories, Functional disability, VIPS task

## Abstract

Visuospatial processing speed underlies several cognitive functions critical for successful completion of everyday tasks, including driving and walking. While it is widely accepted that visuospatial processing speed peaks in early adulthood, performance across the lifespan remains incompletely characterized. Additionally, there remains a lack of paradigms available to assess visuospatial processing speed in unsupervised web-based testing environments. To address these gaps, we developed a novel visuospatial processing speed (VIPS) task adapted from two tests sensitive to visuospatial processing speed declines in older adults, the Useful Field of View paradigm and the PERformance CEntered Portable Test. The VIPS task requires participants to make a central orientation discrimination and complete a simultaneous peripheral visual search task. Data were collected from 86 in-lab volunteers (18–30 years) to compare performance to traditional neuropsychological measures. Consistent with previous literature, performance on the novel VIPS task significantly correlated with measures of selective attention, executive functioning, visual speed, and working memory. An additional 4395 volunteers (12–62 years) were recruited on TestMyBrain.org to establish lifespan trajectories of visuospatial processing speed and associations with functional disability. VIPS task performance peaked in the early 20’s, and steadily decreased such that thresholds doubled in 60-year-olds relative to 20-year-olds (817 ms vs. 412 ms). VIPS task performance significantly correlated with self-reported cognitive functioning deficits broadly across the lifespan but was specifically related to mobility issues in middle-age. These findings have important implications for early detection of cognitive decline and provide insights into potential early intervention targets for younger and middle-aged adults.

## Significance statement

Computer-based tasks have been shown to be helpful for assessing fall risks as well as impairment in activities of daily living and have been widely used as outcome measures of interventions aimed at enhancing cognition in older adults. The present study introduces a new computer-based measure of visuospatial processing speed sensitive to individual differences at a younger age and demonstrates how it relates to disability across the lifespan. This work is critical for understanding how individuals can be functionally impacted by gradual changes in cognitive ability throughout their lives and provides a potential early assessment tool and insight into targets for remediation of decline.

## Introduction

The capacity to rapidly extract and process visual information is critical for the successful completion of a range of everyday activities, and as a result, substantially influences quality of life. Visuospatial processing speed is a core cognitive function that underlies a variety of other processes, including visual working memory (Brown et al., 2012) and overall executive functioning (Mewborn et al., 2015). Alterations in visuospatial processing speed are associated with reduced task performance across a range of domains in older adults, leading to the theory that many cognitive impairments commonly associated with age-related decline are attributable to deficits in processing speed (e.g., Salthouse, 1991, 1995, 1996, 2004, 2005). Visuospatial processing speed is subject to interference and degradation from multiple neurological and psychiatric conditions, such as depression (Tsourtos et al., 2002), dementia (Yamin et al., 2015), and severe traumatic brain injury (for review see Mathias & Wheaton, 2007). In older adults, deficits in visuospatial processing speed are associated with mobility loss (Killane et al., 2014) and global cognitive decline (Deary et al., 2010; Park & Reuter-Lorenz, 2009) beyond traditional measures of visual sensory function. Visuospatial processing speed has been an active target for cognitive training approaches that seek to improve functional outcomes (Ball et al., 2002; Edwards et al., 2002, 2005a, 2005b; Roenker et al., 2003). However, the association between visuospatial processing speed, more global cognitive functioning, and functional disability has not been well documented in young and middle-aged adults. Thus, while a decline in visual processing speed is well established in older adulthood (e.g., McAvinue et al., 2012; Ruiz-Rizzo et al., 2019), changes that occur across the lifespan remain incompletely characterized. One goal of the present study was therefore to assess trajectories of visuospatial processing speed using performance on a novel remotely administered task, and identify associations with functional disability across the adolescent and adult lifespan.

It has been suggested that older adults’ visual functional difficulties emerge under a particular set of conditions. Specifically, tasks that involve searching a visually cluttered field (e.g., searching a cabinet for a prescription bottle) and dividing visual attention (e.g., avoiding obstacles while driving) are often challenging for older adults to complete, and these impairments are particularly evident when time restrictions are imposed (Ball et al., 1990; Kosnik et al., 1988; Sekuler & Ball, 1986) as these activities require rapid processing and encoding of visual information. These impairments cannot be attributed to general vision loss, as older adults without visual deficits often exhibit the same functional disabilities (Owsley, 2013). This suggests that processing speed during divided attention contributes to disability above and beyond impairments in primary visual functions such as acuity or contrast sensitivity (Wood et al., 2005). While evident in older adults, it is currently unknown if processing speed during divided attention contributes to functional impairments in young adulthood, when these functions tend to peak. That is, it remains unclear whether functional impairment resulting from cognitive deficits, such as deficits in visuospatial processing speed, is age invariant.

The phrase “useful field of view” was originally used to denote the size of space from which meaningful information can be obtained without the need for additional head and eye movements (Ball et al., 1988; Sanders, 1970). However, evidence suggests strong correlations between the eccentricity that stimuli can be efficiently processed within a fixed amount of time and the time required to process the same stimuli at a fixed eccentricity across individuals (Edwards et al., 2005a, 2005b). This insight provided the basis for a computer-based paradigm which manipulates stimulus presentation duration with fixed stimulus eccentricities, called the Useful Field of View task (UFOV^©^). In the UFOV paradigm, stimuli are presented for decreasing intervals until the minimum duration at which participants can accurately report the stimuli displayed is identified and recorded. Task performance is a validated predictor of older adults’ car accident involvement (Anstey et al., 2012; Ball et al., 2006; Friedman et al., 2013; Hoffman et al., 2005; Owsley et al., 1998), activities of daily living performance (Aust & Edwards, 2016; Edwards et al., 2005a, 2005b; Owsley et al., 2002), and mobility impairments (Broman et al., 2004; Owsley & McGwin, 2004; Stalvey et al., 1999; Vance et al., 2006). Due to its sensitivity, the UFOV is frequently used as an assessment of driving abilities in clinical populations, such as Parkinson’s disease (Classen et al., 2009; Crizzle et al., 2012), mild cognitive impairment (Wadley et al., 2021), stroke (George & Crotty, 2010), and traumatic brain injuries (Novack et al., 2006). The UFOV, however, is limited in its generalizability to diverse web-based testing samples. First, the UFOV task has been copyrighted and thus the current design is not readily available for web-based platforms. Second, the UFOV consists of four subtasks with published normative data based on the average of three or four of these tasks. Healthy young adults can achieve the minimum threshold in the first two tasks with ease, while reports from a study including younger adults suggest that Subtest 3, the divided attention condition, does not show floor effects for younger adults (Wouterson et al., 2018). Given the need to minimize testing time to reduce subject attrition rates in online testing environments and previous work suggesting the divided attention aspect of the UFOV relates to functional outcomes (Owsley, 2013), adaptations focusing on the divided attention condition component are the ideal subtask to focus on for web-based characterization of visuospatial processing speed capabilities across a broader age range. Finally, while there is evidence to support the ecological validity of UFOV performance in older adults, (e.g., Aust & Edwards, 2016; Broman et al., 2004; Edwards et al., 2005a, 2005b; Owsley & McGwin, 2004; Owsley et al., 2002; Stalvey et al., 1999; Vance et al., 2006), it has yet to be determined whether the association between visuospatial processing speed and functional status holds when individuals are at their peak processing speed.

The PERformance CEntered Portable Test (PERCEPT; Rosen et al., 2015) is an adapted version of the UFOV that was developed to assess deficits in the functional field of view in patients with glaucoma. The UFOV, although sensitive to changes in the visual field that occur throughout healthy aging, is not well-suited for assessing abilities in clinical populations with visual field loss (Chisholm et al., 2008; Crabb et al., 2004; Rosen et al., 2015). To enhance sensitivity for detection of peripheral and perifoveal visual field impairments in individuals with glaucoma relative to the UFOV, the PERCEPT uses a dual-task approach. In this design, the central task involves identifying the orientation of a tumbling E. This is an orientation discrimination task of a capital E presented in sloan font oriented in one of 8 equally spaced rotations. The peripheral task requires identification of the location of a vertically oriented Gabor patch, which is presented in one of 8 radial locations at a fixed 7.7 deg eccentricity. Studies using the PERCEPT suggest that it can successfully identify individuals with glaucoma that have a history of falls and car crash involvement (Rosen et al., 2015). These results suggest that the central/peripheral dual discrimination task features of the UFOV and PERCEPT paradigms, rather than the specific stimuli used, are the critical aspects that allow these measures of visuospatial processing speed to relate to functional outcomes. Additionally, while the use of iPads allows for implementation of high-frequency Gabor stimuli in the peripheral task of the PERCEPT, this makes the paradigm more sensitive to display specifications, such that differences in device resolution and/or luminance could undermine the reliability of the assessment and standardization of testing conditions is therefore required.

Remotely administered assessments introduce significant constraints, including the inability to control an array of conditions that alter display properties. For instance, stimulus luminance is impacted by the brightness settings of monitors as well as environmental lighting situations (e.g., sunlight can cause variable amounts of glare on a monitor depending on the time of day). Additionally, stimulus displays and eccentricities can be standardized across in-lab subjects, however, in a web-based testing environment this is not the case. In fact, unsupervised remote testing will result in differences in display properties and testing at different eccentricities due to variations in the display size and distance of the observer. While programs can query the pixel resolution of a monitor, the physical dimensions of pixels vary depending on screen size and the resulting visual eccentricities of stimuli further depend on the distance of an observer. As a result, validated, fast, and reliable measurements of visuospatial processing speed developed to date are either not readily available, or necessarily suitable, for web-based testing. As such, alternative assessments of visuospatial processing speed are necessary to capture changes in ability across the lifespan in settings where participants may not have access to the same types of display devices and observer distance cannot be monitored.

We have recently developed the VIsuospatial Processing Speed (VIPS) task, a novel visuospatial processing speed task that incorporates a central and peripheral dual-task design adapted from the UFOV (Ball & Owsley, 1993) and the tumbling E central task used in the PERCEPT (Rosen et al., 2015). Our goals in developing the VIPS task were twofold. First, our primary motivation was to develop a paradigm that would be more robust to display variations, a critical requirement for tasks that are hosted on web-based testing platforms. Specifically, we wanted to design a fast and reliable task that was not readily subject to interference based on differences in display properties and observer characteristics. While the PERCEPT involves detection of a single low-contrast grating in the peripheral task, the UFOV involves simple visual search tasks requiring localization of a car-shaped object amongst an array of triangle-shaped distractors, and as such is less dependent on specific device properties. Second, we sought to increase task difficulty in such a way that reliable threshold estimates could be measured in 5 min or less across a large age-range, capturing peak performance in young adulthood (as in Fortenbaugh et al., 2015; Hartshorne & Germine, 2015) while also being able to assess age-related declines in older adults. To achieve the two goals outlined above, we used a visual search as the peripheral task that requires participants to identify a blue triangle amongst a ring of red triangles and red or blue diamond distractors. The incorporation of the color/shape visual search task, similar to classic conjunction search tasks, was intended to increase peripheral task difficulty, as they are known to require more attentional engagement than feature searches (Carrasco et al., 2006; List et al., 2008; Treisman & Gelade, 1980; Treisman & Sato, 1990; Wolfe et al., 1989). To reduce total task time, we further implemented an adaptive threshold estimation using the bestPEST approach (Bach, 1996). Employing these characteristics equipped us to examine the trajectories of visuospatial processing speed throughout the lifespan and capture potential transitions in performance that may occur throughout younger and middle adulthood. As studies assessing the relationship of processing speed with cognitive domains and functional outcomes with the UFOV have involved samples of older adults (e.g., Aust & Edwards, 2016; Broman et al., 2004; Edwards et al., 2005a, 2005b; Matas et al., 2014; Owsley & McGwin, 2004; Owsley et al., 2002; Stalvey et al., 1999; Vance et al., 2006), the present study sought to address two questions. First, in a young adult sample, we designed a task optimized for web-based administration and examined the association between task performance and the cognitive domains typically implicated in these central/peripheral dual-task paradigms using standard neuropsychological measures. Second, we employed this task in a large web-based sample and sought to characterize lifespan trajectories of visuospatial processing speed on this task while also examining the extent to which associations between visuospatial processing speed and functional disability are age-invariant.

## Experiment 1

In the first experiment, we introduced the VIPS task, a novel paradigm intended to assess visuospatial processing speed in a sample of healthy young adults to establish its sensitivity to individual differences at peak ability and potential for remote administration. Previous work has provided evidence that visuospatial processing speed underscores many core cognitive functions including visual attention, executive functioning, visual speed, and visual memory (Anstey et al., 2012; Edwards et al., 2006; Lunsman et al., 2008; Matas et al., 2014; Woutersen et al., 2017). This association may, in part, be due to the fact that tasks designed to tap into the aforementioned constructs are inherently dependent upon visuospatial processing speed. For instance, many of the measures contain a time constraint (e.g., Trail Making Task) or performance is based on time to completion (e.g., RSAT). As such, high performance on these tasks depends on individuals’ ability to rapidly process visually presented information. Similarly, measures of memory, such as the Brief Visuospatial Memory Test-Revised (BVMT-R; Benedict, 1997) and Rey–Osterrieth Complex Figure Test (ROCF; Osterrieth, 1944; Rey, 1941), critically depend on the ability to efficiently process and encode visual information. Therefore, as measures of many of these constructs appear to be intertwined, we hypothesized that the VIPS task would be correlated with traditional neuropsychological measures of these related processes to a similar extent as that previously observed in studies using the UFOV task.

In the current study, we compared VIPS task performance to a series of traditional neuropsychological measures and examined whether this assessment of visuospatial processing speed was significantly associated with the same cognitive domains in young adults as previously demonstrated in older adults (i.e., attention, executive functioning, visual speed, and memory; Anstey et al., 2012; Edwards et al., 2006; Lunsman et al., 2008; Matas et al., 2014; Woutersen et al., 2017). We hypothesized these associations would be inherent to visuospatial processing speed rather than a result of aging and would therefore be present in a sample of healthy young adults at peak processing speed ability.

### Methods

#### Participants

Data were collected from 86 participants who were enrolled in a larger behavioral study that was designed to develop novel web-based assessments of visual and cognitive functioning. For the current study, 45 participants completed one battery of assessments while the other 41 participants completed a second battery of assessments. All participants completed a core set of neuropsychological assessments with three-to-four additional behavioral tests also completed during the 2-h testing session. Results from these additional assessments are not reported here. Sample sizes above 40 participants are generally assumed to be required for studies of individual differences with moderate effect sizes (Cohen, 2013). Thus, a planned recruitment of at least 40 participants per battery was determined prior to the study onset. All participants reported in the present study, completed the VIPS task along with a set of standard neuropsychological assessments reported below. Using G*Power 3.1 (Faul et al., 2007, 2009), we determined the sample size needed to achieve 80% power with α = 0.05 for the lowest associations seen between cognitive variables (attention, executive function, processing speed, and global cognition domains) and UFOV performance in the experimental study conducted by Matas et al., 2014 and the meta-analysis completed by Woutersen et al., 2017. Given the minimum bivariate correlation reported of *r* = 0.36, a minimum sample size of 55 was calculated to observe similar effect sizes in the current study. Recruitment took place through advertisement in the greater Boston, MA metropolitan area and targeted individuals 18–45 years of age with normal vision and no documented neurological or cognitive impairments. Data collection took place between 2019 and 2020. The study protocol was approved by the Department of Veterans Affairs (VA) Boston Healthcare System Institutional Review Board. All participants provided informed consent before study procedures and were compensated $15 per hour for their time.

Of the 86 participants enrolled, 9 participants were missing data on one or more of the neuropsychological assessments due to technical difficulties during the gradCPT (*n* = 2) and experimenter error during administration of symbol coding (*n* = 3) and the RSAT (*n* = 6). All available data was used in analyses. Participants were between the ages of 18 and 30 years (*M* = 21.49, *SD* = 2.62). Consistent with reporting guidelines at the time, participants had the option to select male or female, or elect not to respond for their gender identity. While we recognize that gender is not a binary construct (and data from Experiment 2 collected at a later date included a non-binary option), all participants chose to respond and the sample self-identified as 26.74% male and 73.26% female. Participants were given the option to report their race using NIH reporting guidelines and were given following options to select from: Caucasian, Asian/Pacific Islander, African American, and Latino. Multiple selections were accepted and are reported below accordingly (Table 1). Participants were also given the option to report ethnicity and results are described in Table 1. The sample was largely Caucasian (50.00%) and non-Hispanic or Latino (88.37%). All participants passed visual screening assessed by the FrACT (Bach, 1996) and reported no history of neurological or cognitive impairments.

**Table 1:** Final sample participant demographics

Sample size	Mean	SD
	*N* = 86	
Age (years)	21.49	2.62
Male (%)	26.74	
Education (years)	15.54	2.17
*Race (%)*		
Caucasian	50.00	
Asian/Pacific Islander	24.44	
African American	12.79	
Latino	6.98	
Asian/Pacific Islander, Caucasian	2.33	
African American, Caucasian	1.16	
Caucasian, Latino	1.16	
Chose not to respond	1.16	
*Ethnicity (%)*		
Non-Hispanic or Latino	88.37	
Hispanic or Latino	10.47	
Chose not to respond	1.16	

Race and ethnicity reflect the options exactly as listed following NIH reporting guidelines. Those who reported being biracial did so by selecting multiple options from the given list. One participant elected not to report their race or ethnicity

### Materials and procedures

**Fig. 1:**
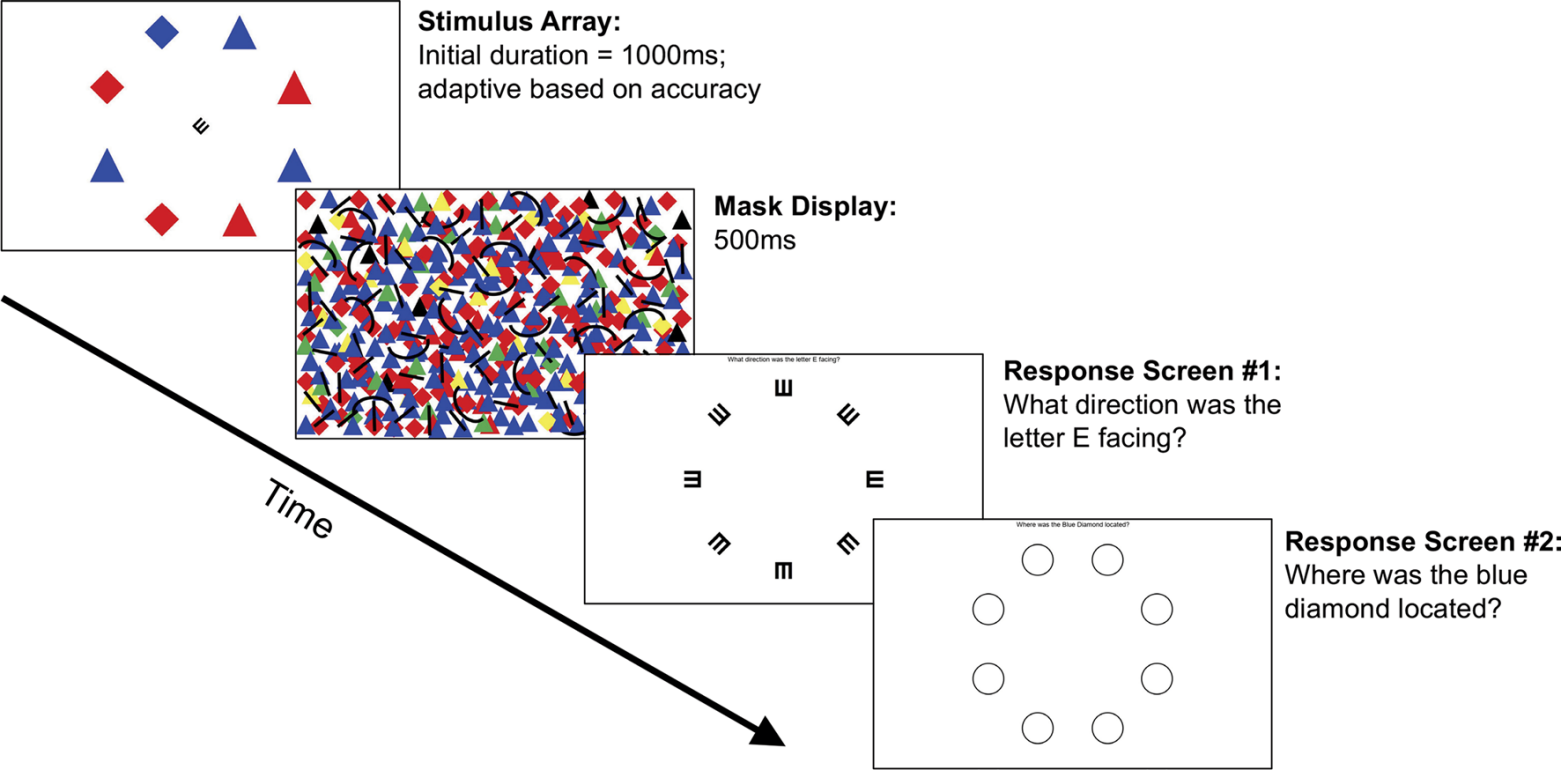
The VIPS paradigm. Schematic illustrating the trial structure with initial stimulus display, mask, and two response screens. Participants used a mouse to indicate the perceived orientation of the central tumbling E and then the location of the blue diamond peripheral target. The use of bright colors along with simple shapes allows the task to be relatively invariant to device and display differences

*VIsuospatial Processing Speed (VIPS) Task* The present study included a novel VIsuospatial Processing Speed (VIPS) task (Fig. 1) that employed a central and peripheral dual-task design derived from the Useful Field of View test (UFOV; Ball & Owsley, 1993) and the PERformance CEntered Portable Test (PERCEPT; Rosen et al., 2015), but with several distinct adjustments. These tasks have been shown to effectively measure processing speed and have demonstrated associations with functional disability (e.g., Aust & Edwards, 2016; Owsley et al., 2002), which therefore motivated our implementation of a similar dual-task design. For the central task, participants were required to discriminate the orientation of a tumbling E. The tumbling E is a simple orientation discrimination task where individuals identify which direction a capital E presented in Sloan font is facing. It can be oriented to one of 8 equally spaced rotations (as in Rosen et al., 2015), and is widely used to assess acuity thresholds, as it does not require individuals to be literate. This functions as a fairly easy central task to help ensure fixation was initially maintained at the center of the screen. The orientation of the tumbling E had to be correctly completed for the threshold to be adjusted. The peripheral task was a conjunction-like search that required participants to identify the location of a blue diamond among an array of distractors consisting of red diamonds and red or blue triangles. We chose opposing red/blue color pairs to promote task invariance for individuals with potential color blindness (i.e., red-green and yellow-blue). Additionally, we avoided colors close in hue (e.g., red–orange) that may be more susceptible to display differences across devices. The use of a color/shape conjunction-like task was employed to help make the paradigm more invariant to display resolution differences and robust to different platforms, and therefore better suited for remote web-based administration than complex objects with high spatial frequency features. Specifically, web-based administration opens potential interference from different testing environments including, but not limited to, external lighting on the monitor, monitor size, and monitor brightness.

As the primary consideration was reliability in an unsupervised testing environment, simple shapes were selected over complex shapes because they are less likely to be impacted by display variations like resolution and environmental lighting. While multiple shape combinations were tested during pilot testing, we sought to include shapes that would be easily known by the broadest demographic to ensure that familiarity with a shape would not impact results (e.g., diamond versus hexagon). With the diamond/triangle combination we further found during the task development phase that two additional features prevented younger adults piloting the task from reaching the minimum threshold. These were (1) inclusion of a mask consisting of multiple colors including the target/distractor colors and black lines and curve shapes oriented in multiple directions to prevent afterimages on the computer screen highlighting the location of the blue objects. (2) Rotating the location of the peripheral shapes by 22.5 deg from the orientation of the tumbling E stimuli. This prevented the tumbling E at the center of the screen from ever “pointing at” the direction at the target shape. While observer viewing distance was not controlled with a chinrest during the experiment as fixed viewing distances would not be possible in web-based testing sessions at a later time, for the present experiment stimuli were always presented on a 15″ Macbook Pro (1440 × 900 screen resolution). Assuming a viewing distance of 60 cm, the central tumbling E was 1.8° × 1.8° centered at fixation while the distractor shapes were 2.5° × 2.5° in size centered at 9.0° eccentricity.

Task difficulty was modified through staircasing stimulus presentations, which was controlled by a 50-trial duration best parameter estimation by sequential testing (bestPEST) adaptive algorithm, assuming a constant fixed slope of two for the psychometric function (Bach, 1996). Initial stimulus durations began at 1000 ms and adapted within a range of 16–2000 ms. Implementation of the bestPEST allows us to acquire more fine-grained estimates of individual differences and age trends. Specifically, we obtain 124 potential outcome thresholds in 50 trials, as opposed to the traditional 2% step change in resolution associated with accuracy measures. A 500 ms mask display screen consisting of brightly colored shapes in a random overlapping display followed the stimulus array to increase task difficulty by preventing the use of afterimages against the otherwise-white screen background as cues to inform participant responses. In this task, the bestPEST determines the stimulus duration (*threshold*) that is required for a participant to maintain 56.25% accuracy (Bach, 1996). This accuracy threshold was selected based on the 8 alternative forced choice peripheral task. The central fixation task must be accurately completed in order for the trial to be considered correct and have peripheral task accuracy calculated. If the central fixation task is incorrect, then the trial is considered inaccurate. Lower stimulus thresholds reflect better task performance for an individual. Following the approach used in the Freiburg Visual Acuity & Contrast Test (FrACT; Bach, 1996), on every 8th trial, participants were presented with an “easy” trial in which durations were 3-times the individual’s estimated stimulus threshold. The intermittent “easy” trials and limited task length (< 5 min) were used to minimize attrition rates, which is critical for remote assessment. For all participants, the VIPS task was administered at both the beginning and end of the assessment protocol in order to assess intrasession test–retest reliability. We found that participants performed consistently across assessments (*r* = 0.81), demonstrating good task reliability.

*The Gradual Onset Continuous Performance Task (gradCPT)* The gradCPT (Esterman et al., 2013) is a well-validated assessment of sustained attention with established lifespan trajectories (Fortenbaugh et al., 2015; Park et al., 2021; Wooten et al., 2019) and sensitivity to neuropsychiatric impairments (Auerbach et al., 2014; Dutra et al., 2018; Esterman et al., 2019; Evans et al., 2022; Fortenbaugh et al., 2017; Kucyi et al., 2020; Mitko et al., 2019; Rothlein et al., 2018). Every 800 ms gray-scale scenes gradually transition from one to the next to eliminate the capture effects of abrupt stimulus onsets, so task performance reflects intrinsic sustained attention ability rather than exogenous alerting. In the gradCPT, participants make frequent responses to city scenes (90% of stimuli) and withhold their responses to rare mountain scenes (10% of stimuli). For the current study, participants completed the validated 4-min version of the task (Fortenbaugh et al., 2015). The primary measure of sustained attention is task accuracy (*d*′; Fortenbaugh et al., 2015), which is calculated using signal detection theory and is based on miss rate, reflected by a failure to respond to cities (omission errors), and false alarm rate, or a failure to withhold responses to mountains (commission errors). Other primary outcome measures from the task are reaction time (RT), reaction time variability (CV), and criterion, which are considered in the analyses below.

*Neuropsychological Assessments* In addition to the VIPS task and gradCPT, participants completed a comprehensive neuropsychological battery to determine which cognitive domains were most closely associated with VIPS task performance. The Freiburg Visual Acuity & Contrast Test (FrACT; Bach, 1996) was administered as a screening measure for visual impairment. Consistent with previous work (i.e., Woutersen et al., 2017), we examined the domains of attention, executive functioning, visual speed, and memory. We used the following neuropsychological measures: the Trail Making Test parts A and B which measures visual speed and executive functioning (TMT; Spreen & Strauss, 1998), the Digit Span Sequencing and Digit Symbol Substitution subtests from the Wechsler Adult Intelligence Scale-III which measure working memory and processing speed (WAIS-III; Wechsler, 1997), and the Ruff 2&7 Selective Attention Test (RSAT; Ruff et al., 1986). Several scores can be derived from the RSAT, including the automatic detection scores, which are calculated from trials that require identification of targets categorically different from distractors, measuring more of what is considered automatic attention capture. Alternatively, the controlled search scores are calculated from trials where the target and distractors require within-category distinctions, reflecting conditions similar to a conjunction search, and controlled attention (Ruff et al., 1986; Schneider & Shiffrin, 1977). Additionally, difference scores can be determined by subtracting controlled scores from automatic (i.e., Automatic—Controlled) and can be used as indices of selective attention. Greater differences between automatic and controlled scores indicate a greater deficit in controlled selective attention. In the present study we considered both the automatic detection and controlled search scores as primary outcome measures for the RSAT, and we further considered discrepancy scores. Two different visual memory tasks were used across sets of participants, such that the first 45 participants completed the Brief Visuospatial Memory Test-Revised (BVMT-R; Benedict, 1997) and the other half (*n* = 41) completed the Rey–Osterrieth Complex Figure Test (ROCF; Osterrieth, 1944; Rey, 1941) to assess memory. As several participants performed at ceiling levels on the BVMT-R, after consultation with a neuropsychologist, the ROCF test was substituted in to provide a more challenging memory task for the young, neurologically healthy sample.

*Statistical Analyses* To assess which cognitive domains were associated with the VIPS task, we compared task performance with traditional neuropsychological measures using a series of bivariate correlations. Based on previous literature (see Woutersen et al., 2017), we hypothesized that VIPS task performance would be associated with measures of selective attention, executive functioning, visual speed, and working memory, and accordingly examined performance on neuropsychological tests that assessed these domains.

### Results

We found significant associations between VIPS task performance and the expected cognitive domains established in previous meta-analyses (Woutersen et al., 2017). VIPS task performance was significantly correlated with attention: gradCPT *d*′, RSAT Controlled Search Accuracy, RSAT Controlled Search and Automatic Detection Speed difference score, RSAT Accuracy, and Digit Span Forward; executive functioning: TMT-B; visual speed: TMT-A; and working memory: Digit Span Backward, and the BVMT-R learning score (Table 2). There were no significant associations between task performance and any of the other measures within these domains (all *p*-values > 0.05).

**Table 2:** Correlations between VIPS task performance and assessments of attention, executive functioning, visual speed, and memory

Domain	Assessment	Pearson’s *r*	*p-*value	*q*-value
Attention	gradCPT *d*′	− 0.22*	0.04	0.09
	gradCPT mean RT	0.20	0.07	0.12
	gradCPT RT variability (CV)	0.18	0.11	0.17
	gradCPT criterion	− 0.12	0.28	0.37
	RSAT automatic detection speed^a^	− 0.01	0.96	0.96
	RSAT automatic detection accuracy^a^	− 0.17	0.12	0.17
	RSAT controlled search speed^a^	− 0.21	0.07	0.13
	RSAT controlled search accuracy^a^	− 0.37***	< 0.001	0.02
	RSAT speed difference^a^	0.28*	0.01	0.03
	RSAT accuracy difference^a^	0.31**	0.005	0.03
	Digit span forward	− 0.27*	0.01	0.03
Executive Functioning	TMT-B	0.32**	0.002	0.01
Visual Speed	TMT-A	0.36***	< 0.001	0.01
	Digit symbol substitution	− 0.21	0.06	0.12
Memory	Digit span backward	− 0.28*	0.01	0.04
	BVMT-R delayed recall	0.08	0.60	0.71
	BVMT-R learning	0.35*	0.02	0.05
	ROCF copy	− 0.03	0.86	0.91
	ROCF immediate recall	− 0.10	0.54	0.68
	ROCF delayed recall	− 0.07	0.67	0.74

^a^denotes Spearman Rho, * denotes *p* < 0.05; ** denotes *p* < 0.01; *** denotes *p* < 0.001. Note that *q*-values show false-discovery rate *p*-values corrected for multiple comparisons

The present analyses were designed to replicate the associations previously observed in the meta-analysis (Woutersen et al., 2017); however, we took a more conservative approach and completed FDR-corrected correlations. Even with these more stringent thresholds, we found that VIPS task performance significantly correlated with attention, specifically selective attention: RSAT Controlled Search Accuracy (*q* = 0.02), the difference between Controlled Search and Automatic Detection Speed (*q* = 0.03) and Accuracy (*q* = 0.03); executive functioning: TMT-B (*q* = 0.01); visual speed: TMT-A (*q* = 0.01); and working memory: Digit Span Backward (*q* = 0.04). Associations with the gradCPT *d*′ and the BVMT-R learning score were not significant (*q* = 0.09 and *q* = 0.05, respectively). Taken together, we demonstrate robust associations between VIPS task performance and the cognitive domains selective attention, executive functioning, visual speed, and working memory.

### Discussion

The present study introduced a novel visuospatial processing speed task (VIPS) optimized for remote assessment and compared it to a series of standard neuropsychological assessments of attention, executive functioning, visual speed, and working memory. We found that the adaptive nature of the task enabled us to capture the dynamic range in performance in healthy young adults, when peak processing speed tends to occur in life, and found significant associations that mirror previous findings in older adults showing associations between the UFOV and measures of visual speed and executive functioning (e.g., Woutersen et al., 2017). The associations with performance on the VIPS task and the measures of selective attention derived from the RSAT (i.e., controlled search accuracy, accuracy difference, and speed difference) may also suggest specificity for the relation between visuospatial processing speed and controlled selective attention rather than more global attention abilities. The strength of associations observed between VIPS thresholds and controlled search and discrepancy scores on the RSAT, but not automatic detection scores and gradCPT accuracy, suggest that the VIPS task is tapping into more traditionally characterized selective attention requiring controlled visual search. This pattern is consistent with previous findings relating the UFOV with visual crowding (Matas et al., 2014), which is shown to coincide with attentional resolution (He et al., 1996; Levi, 2008; Whitney & Levi, 2011). Previous work has also demonstrated that performance on the UFOV is associated with change detection performance, which is similar in nature to controlled serial search, where one item at a time is processed to detect a target (Woutersen et al., 2017). This association is consistent with the present finding that the VIPS task taps into more controlled search domains of attention. Additionally, we find associations between VIPS task performance and the BVMT-R learning score, which is consistent with previous work showing that processing speed influences BVMT-R performance in healthy older adults (Tam & Schmitter-Edgecombe, 2013). Although some of our associations are of smaller magnitude relative to work in older adults (Woutersen et al., 2017), to our knowledge this is the first investigation of these associations in individuals at the age of peak processing speed, and a somewhat diminished association is perhaps unsurprising given the performance across multiple domains tested is expected to be at or near maximal performance in a sample of healthy young adults (Hartshorne & Germine, 2015). Additionally, there is significant evidence that performance ranges are greater in older adults, which may inflate the correlations previously documented in those samples (Myerson et al., 2007). Overall, our results are consistent with the findings of previous work with older adults (e.g., Woutersen et al., 2017), and extend our understanding of visuospatial processing speed by providing initial evidence for an age-invariant association between assessments of these cognitive processes and performance on central/peripheral dual task discrimination paradigms. Considering that the present results indicate the VIPS task captures similar cognitive processes in younger and older adults, we implemented the paradigm in a greater sample on the web in order to determine whether the task maintains the same functional relevance as previous measures of visuospatial processing speed and provide additional support for its utility as a remote assessment.

## Experiment 2

In the second experiment, we employed the VIPS task in a large, web-based sample to establish lifespan trajectories of visuospatial processing speed. The ability to process information efficiently underscores the ability to successfully complete a range of tasks. Support for this is provided by previous work demonstrating associations with impaired performance on timed activities of daily living tasks and the UFOV (Owsley et al., 2002). Given an unlimited amount of time, competence may not be a concern, however, in daily life responses are expected to be made within time constraints (Owsley et al., 2002). As such, self-perceived functional disability arises, in part, as a result of inefficiency of cognitive processing (Owsley et al., 2002). As such, we additionally assessed whether VIPS task performance was significantly associated with self-perceived functional disability throughout the lifespan. Since successful completion of the VIPS task requires the selection of targets among distractors, simultaneous processing of competing information, and maintenance of attention, it was expected that particular subdomains of disability may be most affected by these constructs. The current study used the abbreviated World Health Organization Disability Assessment Schedule (WHODAS) as an index of functional disability (Rehm et al., 1999), which has demonstrated associations with cognitive dysfunction and greater disability in the Understanding and Communicating, Mobility, and Participation subscales (Riley et al., 2019). This pattern of results is in line with previous work demonstrating a link between mobility and visuospatial processing speed (Broman et al., 2004; Owsley & McGwin, 2004; Stalvey et al., 1999; Vance et al., 2006), as well as an association between visuospatial impairment and social engagement in older adults (Krueger et al., 2009). Therefore, we anticipate similar associations with the VIPS task and aforementioned subdomains of disability.

Based on previous literature examining processing speed in other visual tasks (Hartshorne & Germine, 2015), we hypothesized that VIPS performance would peak in the late teens/early twenties and decline throughout adulthood. We additionally hypothesized that the VIPS task would have strong associations with particular aspects of self-perceived functional disability in older adults, namely the subdomains of functioning that relate to mobility, social, and cognitive engagement.

### Methods

#### Participants

Data were collected from 4719 online volunteers worldwide through the website TestMyBrain.org. Data collection took place over a span of 4 months, with initial pilot data collection of VIPS task performance in 1369 participants in December 2019. The remaining data collection for 3350 participants took place from January 2020 through April 2020 with the WHODAS 2.0 added to the battery. All participants gave informed consent following the guidelines set by the Harvard University Institutional Review Board before beginning study procedures.

**Fig. 2:**
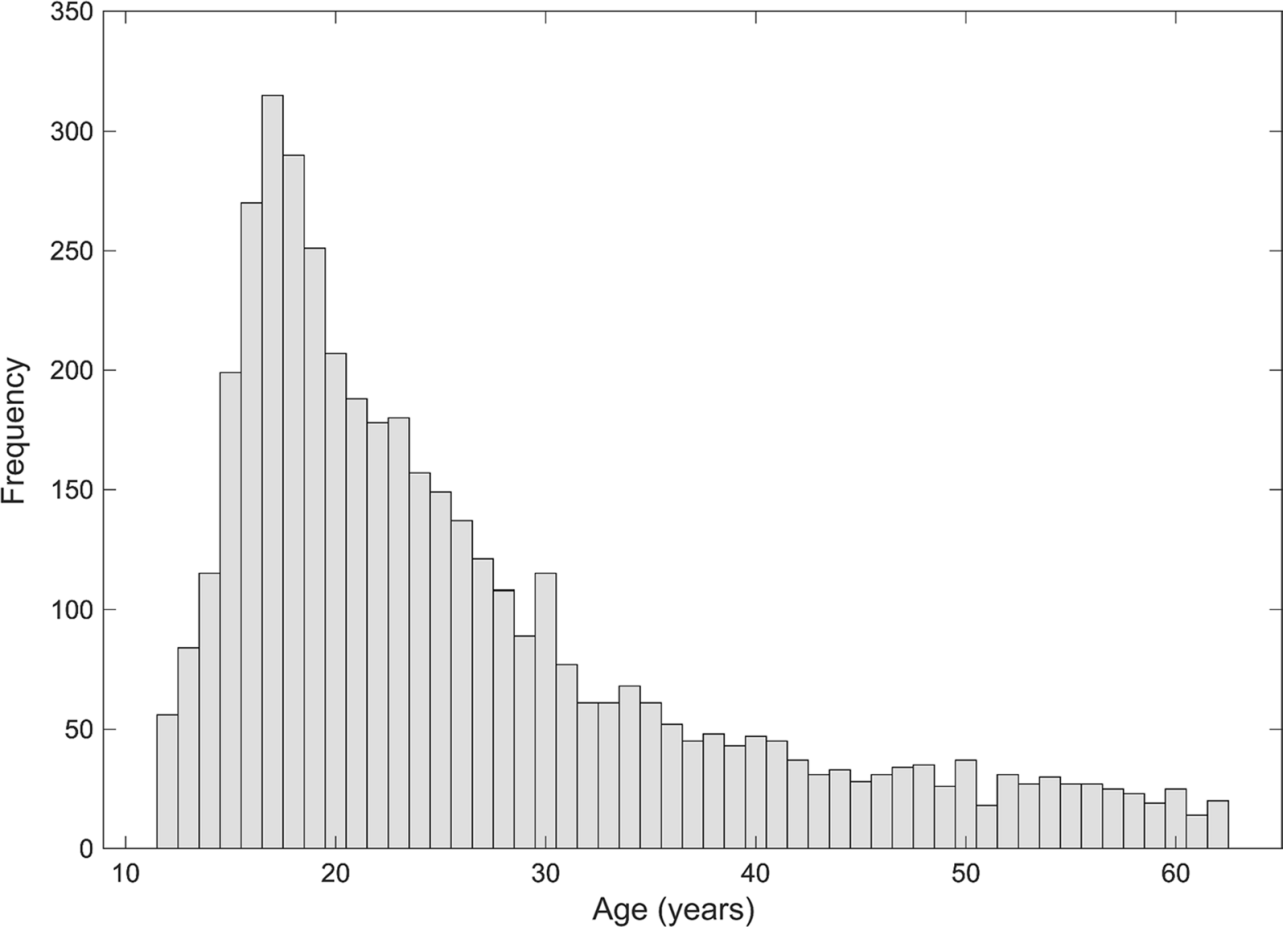
Histogram showing the age distribution of participants for Experiment 2

Of the 4719 participants who completed the task, 324 were excluded, including 90 of those who failed to maintain a predetermined performance threshold (detailed below) and 234 who fell outside age cutoffs. This age range was determined by first determining what ages were adequately represented in order to establish reliable performance estimates (Fig. 2). Given the use of online volunteers, we first determined the range of ages with at least 20 participants for each year. The final sample comprised 4395 participants between the ages of 12 and 62 years (*M* = 26.53, *SD* = 11.79), 59.09% of whom were male, 38.66% were female, and 2.25% were non-binary. Information on race and ethnicity was not recorded in the present sample. The WHODAS 2.0 questionnaire was also completed by 3043 of the subjects who completed the VIPS task.

### Materials and procedures

All participants first completed a short demographic page asking for their age and gender and then completed the VIPS task. For the majority of participants recruited in the last three months of testing, after completion of the VIPS task, participants were also asked to complete the abbreviated World Health Organization Disability Assessment Schedule 2.0 (WHODAS 2.0). The 12-item WHODAS 2.0 questionnaire (Rehm et al., 1999) was administered to assess self-reported functional disability in those who completed the visuospatial processing speed task. The WHODAS evaluates the following six domains of functioning: understanding and communicating, mobility, self-care, getting along, life activities, and participation. Each domain is assessed with two questions with a possible response range from 0 (“none”) to 4 (“extreme/cannot do”), indicating the degree to which any disabilities have negatively impacted specific functions over the last 30 days. Scores for each domain are calculated by summing scores for the two questions in each domain and a total score is calculated by summing scores across all 12 questions. Therefore, higher WHODAS scores reflect higher self-perceived functional disability. In the present study, we examined total scores as well as individual scores across the six subdomains. Of the 3350 participants who had the option, 3043 (91%) completed the WHODAS questionnaire.

*Visuospatial Processing Speed (VIPS) Task* The present study employed the same VIPS task introduced in Experiment 1 (Fig. 1). The design and protocol for the task was largely the same as above, but the task was reprogrammed in JavaScript for presentation on web-based platforms. Additionally, as thresholds cannot exceed the upper bound limit that is predefined as 2000 ms, a performance cutoff of 1900 ms was used to ensure that threshold values captured individual differences in an unsupervised web-based testing setting. This cutoff was established based on simulation studies completed by our laboratory which show that completely random responses would result in thresholds between 1950 and 2000 ms (data not included here). This predetermined cutoff was found to exclude individuals whose threshold values fall outside the upper bound as well as those who may fail to follow directions or randomly respond, as both conditions consistently led to thresholds above 1950 ms in the simulations.

### Statistical analyses

*Task Performance* We used a sliding 3-year window to calculate the mean stimulus threshold as a function of age, similar to previous work (Hartshorne & Germine, 2015), with 95% confidence intervals estimated using bootstrapping with the MATLAB function *bootci*. To quantify how visuospatial processing speed performance fluctuates across the lifespan, we completed hierarchical regression analyses using segmented linear functions (i.e., piecewise regression) to model the average thresholds. Segmented linear functions do not assume symmetric trends and can therefore capture the combinations of changes (i.e., growth, plateau, decay) that may occur across the lifespan. The age range at which transitions in performance are likely to occur can be estimated by the breakpoint between the two linear segments and the confidence intervals around it. These segmented linear functions have previously been used to model changes in sustained attention across the lifespan (Fortenbaugh et al., 2015). In the present study, we compared the linear model to a two-phase segmented function with one breakpoint or transition using extra sum of squares F-tests. Assuming the more complicated, 1-break function provided a better fit, we then planned to test the quality of fit of the 1-break function against a segmented linear function with 2-break points. GraphPad Prism v8.4 (GraphPad Software) was used for model fits and comparisons, as well as estimation of asymmetrical profile-likelihood-based 95% confidence intervals for model parameters (Venzon & Moolgavkar, 1988).

*Performance and Functional Disability* To determine whether there was an overall association between visuospatial processing speed and functional disability, we initially ran a series of correlations between VIPS task performance and self-reported disability indexed by the WHODAS 2.0 questionnaire. To further assess the influence age has on the relationship between these constructs, we next examined how task performance and functional disability associations vary across the lifespan. For this analysis, a sliding 10-year window was used to ensure that at least 150 participants were included for the oldest age bin given the relatively smaller number of participants in the older age ranges. For each age bin, we calculated the correlation between thresholds on the VIPS task and self-reported disability scores on the 12-item WHODAS questionnaire (*N* = 3043) using Spearman Rho correlation coefficients to account for any potential deviations from normality that may arise within a given age bin. Given the use of sliding age bins, in order to estimate the significance of the correlation between functional disability and VIPS task scores, Monte–Carlo permutation testing was completed. For each age bin, WHODAS scores were randomly shuffled and correlated with VIPS task performance across 1000 permutations. The top and bottom 2.5% of the shuffled distribution was then calculated to determine the rho cut-off values corresponding to a Monte-Carlo *p* ≤ 0.05 for each age bin.

### Results

#### Performance across the lifespan

**Fig. 3:**
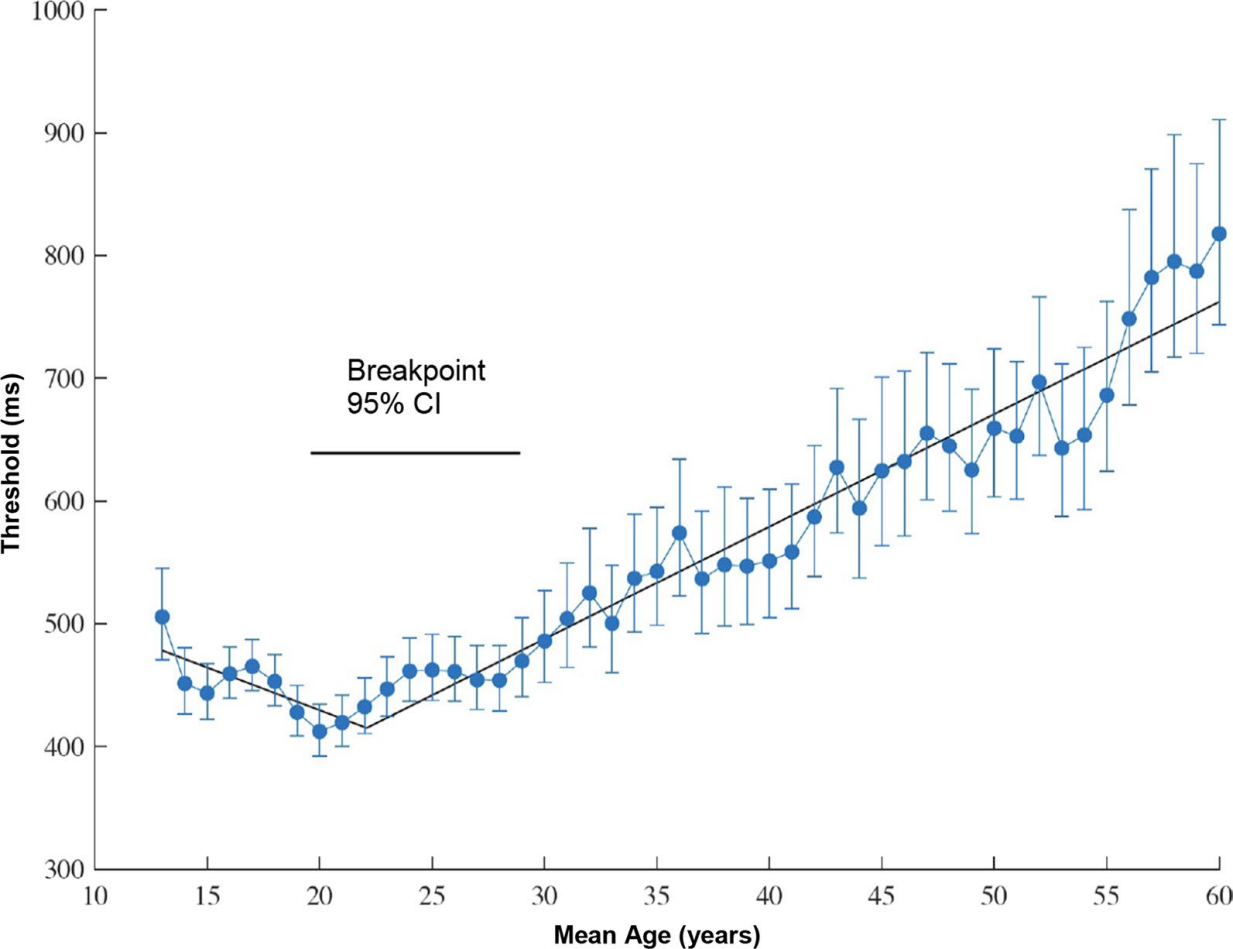
Changes in visuospatial processing speed performance for each age bin. Error bars show the 95% confidence intervals. The trendline depicts the best-fitting function resulting from the hierarchical regression analyses. The estimated breakpoint 95% confidence interval is shown

The present results demonstrate how visuospatial processing speed abilities change across the lifespan (Fig. 3). For the first level model, quality of fits modeling the mean age trends was compared across a straight line versus a segmented linear function with a single break point. Results showed that a 1-break segmented function provided a better fit for the data than the linear model (*F*(2,44) = 29.38, *p* < 0.001; *r*^2^ = 0.95). In the second level model, the single break function model was compared to a 2-break segmented linear function. Results of this analysis showed that the more complex 2-break function did not provide a significantly better fit to the 1-break function (*F*(2,42) = 2.02, *p* = 0.15). Examining the estimated parameters from the 1-break segmented function, we found that VIPS performance peaked at around 22 years (95% CI 19–28), and steadily decreased such that thresholds were doubled in those 60 years of age relative to those 20 years of age (817 ms vs. 412 ms).

#### Association between VIPS task performance and functional disability

Overall, VIPS task performance had a trending association with total WHODAS scores (*r* = 0.03, *p* = 0.08). VIPS performance was significantly correlated with self-reported difficulties in cognitive functioning (Understanding & Communicating subdomain; *r* = 0.08, *p* < 0.001) and mobility (*r* = 0.05; *p* < 0.01). VIPS task performance was not associated with any of the subdomains relating to self-care or psychosocial factors measured by the WHODAS (all *p*-values > 0.05).

**Fig. 4:**
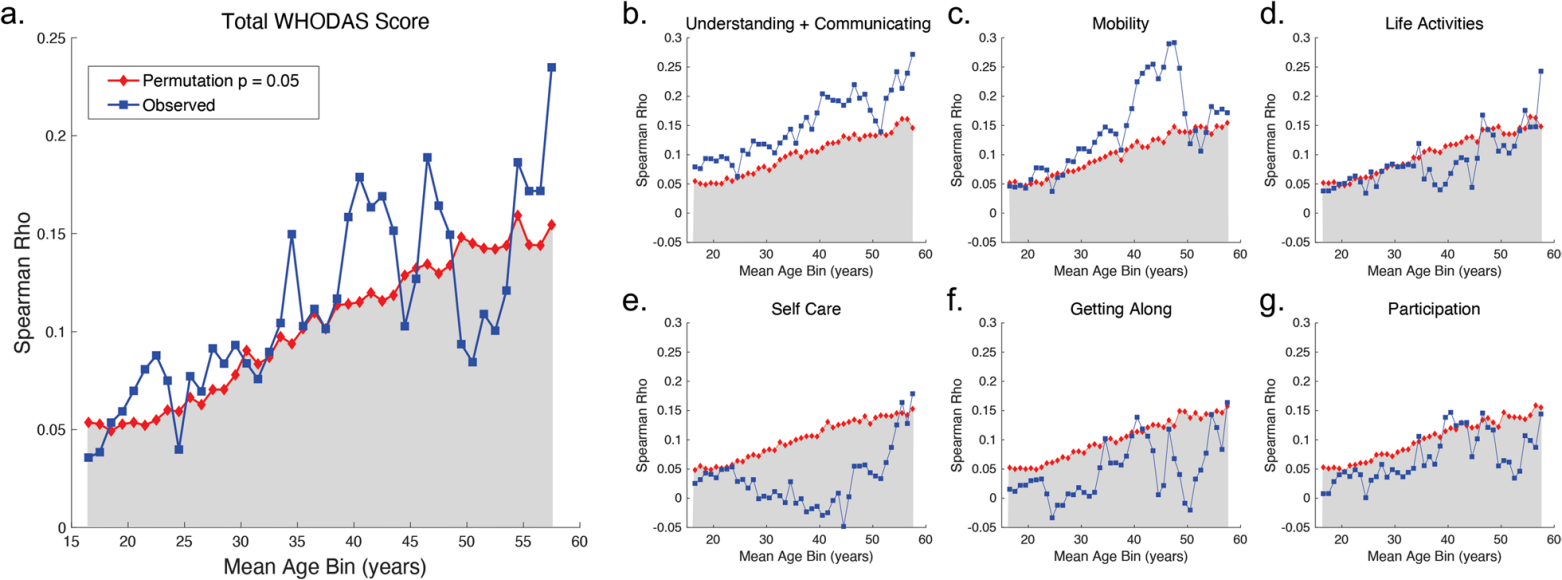
Correlation between VIPs task performance and **a** WHODAS total score and subscores **b** Understanding and Communicating **c** Mobility **d** Life Activities **e** Self Care **f** Getting Along **g** Participation 10-year age bins across the lifespan

Figure 4 shows the results of the correlation analysis between performance on the VIPS task and self-reported functional disability for the different age bins. In each figure, the blue line shows the observed correlation while the red lines correspond to the two-tailed Monte-Carlo minimum rho-value for *p* ≤ 0.05. The shaded areas indicate the rho values that failed to reach significance. When examining the relationship between task performance and functional disability, we again found that task performance significantly correlated with self-reported deficits in cognitive functioning (Understanding & Communicating subdomain) across the lifespan. Task performance remained significantly correlated with mobility issues, particularly for middle-aged participants. In contrast, consistent with our findings examining overall performance irrespective of age, disability related to self-care or psychosocial factors, such as getting along with others, was not found to be significantly correlated with performance on the VIPS task.

#### Comparison of in-lab to online samples

**Fig. 5:**
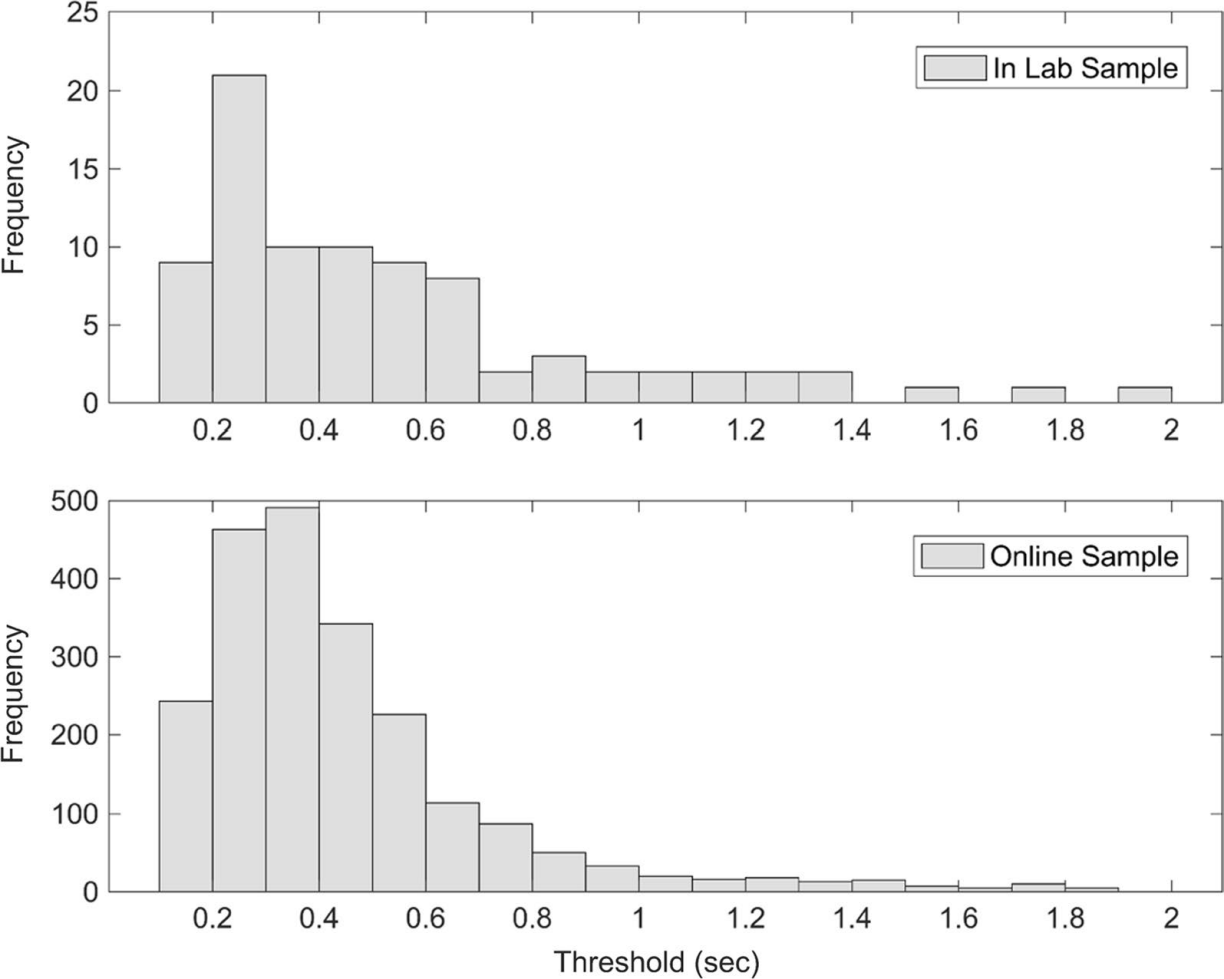
Histograms showing thresholds for all participants between the ages of 18 and 30 who completed the VIPS task in-lab in Experiment 1 (top panel) and online in Experiment 2 (bottom panel)

As a final analysis, we asked whether performance on the VIPS task was similar when completed in a supervised laboratory setting versus online in an unsupervised setting. For this analysis, we compared performance for all 86 participants who completed the VIPS in Experiment 1 with the 2170 participants between the ages of 18 and 30 years who completed the task online through TestMyBrain.org to match the age range of participants in Experiment 1. Figure 5 shows a histogram of individual thresholds for the two samples of participants. Given the differences in sample sizes, a Wilcoxon rank sum test was calculated to compare the median thresholds across groups. No difference was observed in median thresholds across the two samples (in-lab median = 430 ms; online median = 372 ms; *z* = − 1.4996, *p* = 0.1337), suggesting comparable performance distributions for young adults across the two testing platforms.

### Discussion

The present study employed our novel assessment (the VIPS) in a large web-based sample along with a measure of self-perceived functional disability. We found that peak task performance occurred in young adulthood (i.e., early 20 s) and then declined in a linear fashion throughout the lifespan without significant evidence for additional shifts in the rate of change in the older age ranges tested. VIPS task performance was significantly associated with self-perceived deficits in cognitive functioning (Understanding & Communicating subdomain) across the lifespan, and mobility impairments in middle-aged adults. Similar associations have been demonstrated using other assessments of visuospatial processing speed in samples of older adults, such that task performance is predictive of performance on timed instrumental activities of daily living (Aust & Edwards, 2016; Edwards et al., 2005a, 2005b; Owsley et al., 2002), and mobility tasks (Broman et al., 2004; Owsley & McGwin, 2004; Stalvey et al., 1999; Vance et al., 2006). This association likely persists as task time constraints highlight deficits in speed of cognitive processing that likely translate into functional impairment. Additionally, as mobility declines with increasing age, individuals may rely more on cognitive processes like visual sustained attention to compensate for physical limitations (Park et al., 2021). Specifically, visuospatial processing speed aids in the ability to compensate for sensorimotor decline, as navigating complex environments involves rapid extraction and processing of visual information to direct subsequent coordination of movements (Amboni et al., 2013; Yogev-Seligmann et al., 2008). As such, impairments in visuospatial processing speed may be associated with greater functional impairment in mobility, which is consistent with our findings. The present results extend our understanding of these associations, demonstrating consistency in these relationships, with some beginning as early as young adulthood. The lifespan trajectories demonstrate decline in visuospatial processing speed, as assessed with the VIPS task, beginning early in adulthood and further highlight the importance of visuospatial processing speed and functional capacity.

## General discussion

The present study employed a novel task (the VIPS task) to provide critical insight into the lifespan trajectories of visuospatial processing speed. First, we introduced and validated a task designed to assess the dynamic range of visuospatial processing speed across young adults at peak ability, optimized for web-based assessment. Consistent with previous work in older adults (e.g., Anstey et al., 2012; Edwards et al., 2006; Lunsman et al., 2008; Matas et al., 2014; Woutersen et al., 2017), we found that performance was significantly associated with traditional neuropsychological measures of attention, executive functioning, visual speed, and working memory, suggesting an age invariant association amongst assessments of these processes. Our results further suggest that the associations between these processes and visuospatial processing speed that are reported in older adults (Anstey et al., 2012; Edwards et al., 2006; Lunsman et al., 2008; Matas et al., 2014; Woutersen et al., 2017) are likely not the result of global cognitive decline, but rather are inherent to our assessments of visuospatial processing speed, as the relationships hold at peak performance. We then employed the VIPS task in a large web-based sample in order to examine how individuals’ ability fluctuates across the lifespan. Due to the adaptive nature of the task, we were able to examine peak performance in young adults while preserving the sensitivity to individual differences in older adulthood. The trajectories showed that VIPS task performance peaked in the 20 s and steadily declined throughout adulthood. Moreover, task performance predicted self-reported cognitive functioning (i.e., Understanding & Communicating subdomain of the WHODAS) deficits across the lifespan and was strongly associated with mobility difficulties in middle adulthood. The present results have important implications for the role of visuospatial processing speed assessments with respect to functional disability.

Previous work has focused on how visuospatial processing speed declines in older ages and its association with functional performance, specifically driving accidents (Anstey et al., 2012; Ball et al., 2006; Friedman et al., 2013; Hoffman et al., 2005; Owsley et al., 1998) and instrumental activities of daily living (Aust & Edwards, 2016; Edwards et al., 2005a, 2005b; Owsley et al., 2002), and has helped to identify the cognitive processes that are linked to this decline (e.g., Anstey et al., 2012; Edwards et al., 2006; Lunsman et al., 2008; Matas et al., 2014; Woutersen et al., 2017). Findings from these studies have demonstrated significant associations between visuospatial processing speed performance and a series of other cognitive domains (i.e., attention, executive functioning, visual speed, and working memory; Woutersen et al., 2017), specifically in older adults. Previous work has also provided significant evidence that visuospatial functions are uniquely sensitive to age-related decline. Specifically, performance on visuospatial processing speed measures have shown significant age-related slowing effects relative to verbal assessments (Jenkins et al., 2000). Visuospatial abilities have further been shown to differentiate from verbal abilities as working memory performance for visuospatial material decreases more readily across older adulthood (Johnson et al., 2010; Swanson, 2017). In fact, it has been demonstrated that visuospatial memory performance diminishes linearly across the lifespan (Logie et al., 2009) and declines twice as rapidly as verbal memory (Murre et al., 2013). Due to the global decline that accompanies aging, however, it remains unclear if the aforementioned associations between visuospatial processes and other cognitive domains reflect inherent relations between assessments or rather are the result of neural dedifferentiation in older adulthood. The results from the current study shed new light on these relationships, demonstrating that even at peak ability, these assessments of visuospatial processing speed do not appear to measure an isolated process; they seem to be assessing a range of cognitive functions in the absence of any cognitive decline. Regardless, these associations are likely what enables measures of visuospatial processing speed to accurately predict functional outcomes in older adults, as impairment in performance on tasks assessing visuospatial processing speed may more accurately reflect a decline in multiple related processes. Given that visuospatial processing speed appears to be critical for maintaining quality of life, and is particularly vulnerable to age-related alterations, it has been identified as a target for cognitive remediation interventions.

It has been demonstrated across a series of studies that visuospatial processing speed performance, as assessed by the UFOV, is responsive to speed of processing training in older adults (Ball et al., 2002; Edwards et al., 2002, 2005a, 2005b; Roenker et al., 2003). Critically, these effects have been shown to transfer to functional improvements, including driving performance (Roenker et al., 2003) and instrumental activities of daily living (Edwards et al., 2002, 2005a, 2005b). The present results suggest that visuospatial processing speed begins to decline early in adulthood, with performance peaking in the early 20 s and steadily declining thereafter. As the present results replicate the associations typically seen in older adults in a sample of younger and middle-aged adults, it suggests that implementation of targeted visuospatial processing speed training programs earlier in adulthood could have benefits that potentially generalize to real world functional outcomes and improve or prevent the decline from impairing performance of everyday activities (e.g., car accidents while driving; Anstey et al., 2012; Ball et al., 2006; Friedman et al., 2013; Hoffman et al., 2005; Owsley et al., 1998). Given the ease of assessing visuospatial processing speed with the VIPS task and its sensitivity to individual variation in young adults at peak processing speed, it may be feasible to implement the assessment as a screening measure for young adults in order to target these interventions at the beginning of their decline in ability.

The present findings reflecting interdependence between traditional neuropsychological assessments of selective attention, executive functioning, visual speed and working memory and visuospatial processing speed can be interpreted in several ways. The first potential explanation is that each of the processes assessed in the current study involved tasks that are critically dependent upon visuospatial processing speed. That is, all of the aforementioned neuropsychological measures require intact visuospatial processing speed in order to be completed. This interpretation is in line with previous theories assuming that age-related cognitive declines are due to visuospatial processing speed (e.g., Salthouse, 1991, 1995, 1996, 2004, 2005). An alternative explanation, however, is that the cognitive assessments created to evaluate these constructs are not truly process pure. In essence, it is possible that these assessments require individuals to recruit a range of cognitive processes in order to be successfully completed, such as selective attention and working memory. As such, the associations between these domains arise because the tasks intended to assess a single process are in fact assessing multiple cognitive constructs to differing degrees. As these cognitive processes and assessments appear to be inherently confounded, it is unlikely that the best explanation can be discerned. While dissociating cognitive processes is of theoretical interest, it is important to note that most tasks critical to supporting functional independence in daily living (e.g., driving, grocery shopping, etc.) rely on a complex interplay of sensory and cognitive functions. Thus, having readily accessible computer tasks that are sensitive to multiple aspects of visual and cognitive functioning may prove to be better predictors of functional abilities than purer measures of any one cognitive domain.

The current experiments have several limitations that should be addressed. First, the in-lab sample is relatively small (*N* = 86), and the change in the memory task used in half of the participants may have left analyses involving the assessment of memory underpowered. Replication in a larger sample to examine the associations between visuospatial processing speed and related cognitive domains would be beneficial. Additionally, information on race and ethnicity was not recorded for the majority of the sample, so it is unknown whether the participant demographics accurately represent the general population. In the second experiment, we only included data from individuals up to 62 years of age due to limited web-based participation from older adults. The use of this cutoff, however, results in a failure to capture the upper portion of the lifespan with the current sample. Moreover, we cannot determine causality in the association between visuospatial processing speed and self-perceived functional disability with the present cross-sectional work. A longitudinal study is required in order to determine whether a decline in visuospatial processing speed definitively results in functional impairment. Finally, it is important to note that in the second experiment we do not have performance on additional cognitive measures which is necessary to more critically discern the sensitivity of the VIPS task to functional disability and age-related decline relative to other cognitive assessments.

Regardless of these limitations, the present study provides insight into visuospatial processing speed and highlights its functional relevance across the lifespan. We demonstrated a significant association between task performance and the domains of attention, executive functioning, visual speed, and working memory using standard neuropsychological measures. We further characterized the lifespan trajectories of visuospatial processing speed and demonstrated an age-invariant association between visuospatial processing speed and self-perceived disability in cognitive functioning. As the current results mirror findings from previous work examining the UFOV task in older adults, the present study extends our understanding of the cognitive contributions for functional performance and suggests visuospatial processing speed as a potential target for early interventions in both younger and middle-aged adults as well.

## Conclusions

In a sample of healthy young adults at peak ability, VIPS task performance was associated with traditional neuropsychological measures of attention, executive functioning, visual speed, and working memory. In a large, web-based sample, lifespan trajectories of visuospatial processing speed were established, and associations between visuospatial processing speed and specific aspects of self-perceived functional disability were demonstrated. These results suggest that future studies that examine the potential longitudinal benefits of implementing speed of processing screening measures and/or training in younger and middle-aged adults are warranted.

## Data Availability

The datasets presented in this article are restricted under regulations set forth by the United States Department of Veterans Affairs and neither the data nor the materials have been made available on a permanent third-party archive. De-identified data can be made available to eligible researchers. Requests for data and/or materials should be sent via email to the senior author at Francesca_Fortenbaugh@hms.harvard.edu. The VIPS task MATLAB code is freely available to any interested party and should be requested via email to the senior author at Francesca_Fortenbaugh@hms.harvard.edu.

## Abbreviations

bestPEST: Best parameter estimation by sequential testing
BVMT-R: Brief Visuospatial Memory Test-Revised
FrACT: The Freiburg Visual Acuity & Contrast Test
gradCPT: Gradual Onset Continuous Performance Task
PERCEPT: PERformance CEntered Portable Test
ROCF: Rey–Osterrieth Complex Figure Test
RSAT: Ruff 2&7 Selective Attention Test
TMT: Trail Making Test
UFOV: Useful field of view
VIPS Task: VIsuospatial processing speed task
WAIS-III: Wechsler Adult Intelligence Scale-III
WHODAS: World Health Organization Disability Assessment Schedule
